# Qualitative Analysis of the Efficacy of Probiotic Strains in the Prevention of Antibiotic-Associated Diarrhea

**DOI:** 10.7759/cureus.40261

**Published:** 2023-06-11

**Authors:** Nyier W Doar, Shamini D Samuthiram

**Affiliations:** 1 Medicine, Interfaith Medical Center, New York, USA; 2 Medicine, Medical University of the Americas, Charlestown, KNA

**Keywords:** ords: lactobacillus, bifidobacterium, saccharomyces, probiotic, antibiotic-associated diarrhea, prevention

## Abstract

Antibiotic-associated diarrhea is often managed by the withdrawal of the culprit antibiotics or the administration of alternative antibiotics when a *Clostridium difficile* infection (CDI) is suspected, an infection that tends to be the most common causative agent of the disease. Probiotics are also gaining popularity as alternative therapies, and it was hypothesized in this article that a Lactobacillus strain is the most efficacious probiotic for the prevention of antibiotic-associated diarrhea.

This article conducted a literature review investigating the relative efficacy of the Lactobacillus, Bifidobacterium, and Saccharomyces probiotic strains as effective alternative therapies for antibiotic-associated diarrhea. The literature searched was from the PubMed database. The inclusion filters were: random control trials (RCTs), clinical trials, meta-analysis, last 10 years, full-text articles available in English, and all articles published in peer-reviewed journals.

All three probiotic genera had strains that demonstrated significant efficacy in the prevention of antibiotic-associated diarrhea. However, *Saccharomyces boulardii* I-745 tends to outperform all the strains as the most effective and the one with the fewest, if any, adverse effects.

Whenever probiotics are considered for the prevention of antibiotic-associated diarrhea (AAD) in both pediatric and adult patients, *S. boulardii* I-745 should probably be prioritized.

## Introduction and background

Foods consumed in the West are mostly processed and sterile, depriving them of important gastrointestinal commensals and thus predisposing many people to gut-related disturbances [1]. In contrast, food products such as fruits that are consumed in third-world countries are mostly fresh and packed with various commensal microorganisms that are established in the gut, inhibiting the unhealthy symptoms experienced in the West. The use of antibiotics, most commonly ampicillin and clindamycin, even worsens these gut disturbances [2]. Lukasik and Szajewska [3] specifically defined antibiotic-associated diarrhea (AAD) as diarrhea (at least three loose/watery stools per day for at least two days) that occurs during the administration of antibiotics or at least four to eight weeks post-antibiotic discontinuation. About one-third of these AADs are attributed to *Clostridium difficile* infection (CDI), currently the number one cause of healthcare-associated infections (HAIs) in North America [4]. AAD may lead to electrolyte imbalances, pseudomembranous colitis, and toxic megacolon, among other less common presentations.

Current standard treatments for AAD revolve around the use of alternative antibiotics, mostly metronidazole and sometimes oral vancomycin, when the discontinuation of previously used culprit antibiotics does not resolve the AAD [5]. These alternative antibiotics are used to control a suspected CDI, which is responsible for the majority of the AADs, as stated above. Relapse tends to be the major shortcoming of this treatment modality, warranting the use of yet another antibiotic, fidaxomycin [5]. Fidaxomycin is, however, expensive and does not guarantee complete remission. Fecal microbiota transplant has been used to treat relapsing AAD, but Mullane [5] pointed out many limitations of this modality, similar to those associated with the use of fidaxomycin.

Instead of fecal microbiota transplant, probiotic co-administration during antibiotic exposure is currently being employed to prevent AAD [6]. Selinger et al. [6] described probiotics as ingested, non-pathogenic living microorganisms that colonize and restore the microbiota of the intestines. According to Ghasemiana et al. [7], the medical use of probiotics started in the early 1900s, when Elie Metchnikoff won the Nobel Prize with the finding that consumption of yogurt containing Lactobacillus led to a decrease in toxin-producing microbes in the small intestine, increasing the longevity of the host. Since then, the number of studies on probiotics has steadily increased around the world. The probiotics are not only used to prevent AAD but are also commonly used to treat: lactose intolerance, several types of cancer, inflammatory bowel disease, obesity, allergies, travelers’ diarrhea, infant colic, necrotizing enterocolitis, *C. difficile* infection, *Helicobacter pylori* infection, and vaginal yeast infections [7].

There are many types of probiotics that have been found to be effective in the prevention of AAD, which presents the challenge of selecting the appropriate kind for a particular type of disease. A network meta-analysis of 51 random control trials (RCTs) by Cai et al. [8] identified three types of probiotic genera that have demonstrated significant efficacy and, thus, are most commonly used: Lactobacillus, Bifidobacillus, and Saccharomyces. McFarland et al. [9] pointed out that prevention of AAD or other closely related diarrheas like CDI and nosocomial infections from these genera is strain-specific but did not give any insights on the relative efficacy of the specific probiotic strains. The two goals of this paper are, at any given formulation (e.g., yogurt, capsule, etc.) and dosage, to (1) evaluate the evidence for the efficacy of the three probiotic genera (Lactobacillus, Bifidobacterium, and Saccharomyces) for the treatment of the AAD and (2) qualitatively analyze the relative efficacy of the most promising strains from these genera.

## Review

Methods

Search Strategy

The main database searched was PubMed. Other databases included BioMed Central, Google Scholar, and the Directory of Open Access Journals. The bibliographies of the relevant articles were also reviewed. The key MeSH search text was: (probiotic OR Lactobacillus OR Bifidobacillus OR Saccharomyces) AND (prevention and control) AND (anti-bacterial agents) AND diarrhea. Other terms such as "free" and "full-text" were added to the MeSH search text words when searching Google Scholar and the Directory of Open Access Journals.

Inclusion/Exclusion Criteria

The articles included were randomized controlled trials, clinical trials, observational (cohort/case-control) studies, and meta-analyses. These articles were included only if they referred to at least a specific strain of the three probiotic genera identified above, in addition to the search terms above, and were published in peer-reviewed journals. Additional inclusion criteria included articles that had full text available and were free. Finally, only articles that were published within the last ten years were included. Non-English-published articles were excluded from this review. Also, most review articles were removed, although a few reviews were considered for some background information on the subject matter.

Data Extraction

Articles were screened by title, abstract, and full text if necessary. Eligible articles were compiled into Table 1. The relevant data extracted from the articles were organized into five evidence table headings that illustrated the name of the article; the year of the article's publication; the design of the study; the characteristics of the population/subjects studied, and the outcome(s) of the study. Generally, the study population comprised all the patients diagnosed with antibiotic-associated diarrhea. To avoid the inclusion of duplicated data, the names of the authors were thoroughly inspected.

**Table 1:** Evidence table, summarizing the characteristics of the main articles analyzed in this systemic review

Article	Design	Study population	Results/outcome
Cai et al. [8]	Meta-analysis	51 RCTs comparing 10 probiotic interventions.	On prevention of AAD, *L. rhamnosus* GG (LGG) had the highest probability of being ranked best both in effectiveness (odds ratio (OR)), 95% confidence interval (CI). 0.28 (0.17, 0.47)) and tolerance (0.44 (0.23, 0.84)). *L. casei* also had better efficacy (0.04(0.00, 0.77)) in reducing CDI rate.
Ripert et al. [10]	Meta-analysis	Studied the ability of the compounds secreted by the probiotic *B. clausii* to counteract the toxins produced by two pathogens: clostridium difficile and *B. cereus*	The probiotic formulation containing these three Lactobacilli strains (*L. acidophilus* CL1285, *L. casei* LBC80R, *L. rhamnosus* CLR2, Bio-K) is the most efficacious probiotic combination.
Videlock et al. [11]	Meta-analysis	4138 patients from 34 RCTs	Significant prevention of AAD in the probiotic group versus the placebo at a pooled RR of 0.53 (95% CI 0.44-0.63)
Fox et al. [12]	RCT	Children were randomly given 200 g/day of either yogurt containing *L. rhamnosus*, *B. lactis*-12, and *L. acidophilus* La-5 or placebo	No evidence of severe diarrhea in the probiotic group and 6 in placebo.
Sampalis et al. [13]	RCT	214 randomized to Bio-K and 221 to placebo in a hospital setting	Incidence of diarrhea was 21.8% in Bio-K + CL1285 group versus 29.4% in the placebo group, adjusted OR=0.627, p=0.037, showing significant efficacy of L. acidophilus CL. Mechanisms: modulation of intestinal cytokine production, esp., inflammatory cytokines
Sniffen et al. [14]	Meta-analysis	Analyzed 249 trials that showed evidence for 22 different types of probiotics	*L. casei* DN1114001 had 2 RCTs with significant findings, 0 RCTs with non-significant findings. *S. boulardii* I-745 had 18+ versus 9-
Blaabjerg et al. [15]	Meta-analysis	217 RCTs with 3631 participants randomized to either the *L. rhamnosus* *S. boulardii* treatment group or placebo	Found an incidence of AAD in 8.0% of the probiotic group compared to 17.7% in the control group (RR 0.49, 95% CI 0.36 to 0.66)
Alberda et al. [16]	RCT	32 patients participated	AAD was documented in 12.5% of the probiotic group and 31.3% in the control group, providing evidence of the efficacy of *L. casei* drink
Dietrich et al. [17]	Non-randomized prospective cohort	Two *L. casei* strain drinks were directly compared in 60 patients in an RCT	AAD significantly reduced in the intervention group (6.7% versus 33.3%; p<0.021) that had the *L. casei* DN114001 than the group with the *L. casei* Shirota drink
Szajewska et al. [18]	Meta-analysis	4780 patients from 21 RCTS	*S. boulardii* treatment reduced AAD incidence (8.5% versus 18.7%; RR: 0.47; 95% CI: 0.38-0.57) compared to the placebo
McFarland [19]	Meta-analysis	27 RCTS encompassing 5029 patients	*S. boulardii* had a significant therapeutic efficacy in 84% of the treatment arms in the prevention of AAD (RR=0.47, 95% CI: 0.35-0.63, p <0.001)
Thygesen et al. [20]	Case report	A 79-year-old woman treated with antibiotics and *S. boulardii* (Sacchaflor)	The patient developed fungemia 13 days after treatment
Yun et al. [21]	Prospective cohort	Co-cultured Bifidobacterium with *C. difficile*	The survival rates for mice given *B. longum* ATCC 15707 alone, and with live cells, or dead cells of *B. longum* were 40%, 70%, and 60%, respectively.
Patrone et al. [22]	Prospective cohort without controls	Bacterial enumeration from three batches was carried out by plating techniques	Of the five brand names/commercial products for the *B. clausii* in India and Pakistan, only Enterogermina tends to follow the label claim of efficacy of the *B. clausii* for AAD prevention
Lakshmi et al. [23]	Prospective cohort with no controls	Rats exposed to *B. clausii* for acute toxicity	Showed significant efficacy in *B. clausii* use against AAD and its safety
Chatterjee et al. [24]	RCT without controls	Adults randomized to combined *L. acidophilus* CL and Bifidobacterium spp	AAD incidence in only 10.8% of the group randomized to a combined Bifidobacterium spp and L. acidophilus CL compared to 15.6% in the placebo group (RR: 0.7; 95% CI 0.4-1.2)
Valdés-Varela et al. [25]	Case control	Analyzed the capacity of twenty Bifidobacterium and Lactobacillus strains with *C. difficile*	Compared the efficacy of Bifidobacterium vs. Lactobacillus strains: *B. longum* IPLA20022 showed the highest ability to counteract the cytotoxic effect of *C. difficile*, LMG21717
Valdés-Varela et al. [26]	Prospective cohort	Co-cultured a toxogenic *C. difficile* with 4 Bifidobacterium strains	*B. longum* and *B. breve* were the strains showing a higher reduction in the toxicity of the co-culture supernatants
Cameron et al. [27]	Meta-analysis	Meta-analysis of 249 RCTs	The following strains were recommended for AAD prevention: *S. boulardii* I-745, *L. casei* DN114001, and LaLcLr mix (a combination of *L. acidophilus* CL1285, *L. casei* Lbc80r, and *L. rhamnosus* CLR2) for AAD prevention in any age group
Song et al. [28]	RCT	214 patients were randomized to either a Lactobacillus capsule or placebo for 14 days	Lactobacillus strains prevent AAD through modification of toxin receptors, competition for nutrients, competitive inhibition of pathogen adhesion, and the synthesis of antimicrobial substances

Probiotic Strain Designation

Much of the published literature does not refer to a specific strain since strain designations tend to vary by geographic region. Many authors end up resorting to species or generic names of the probiotics, such as *Lactobacillus rhamnosus* or Lactobacillus, respectively, without necessarily referring to specific strain names such as *L. rhamnosus* GG. This systemic review only used the most commonly indicated probiotic strains in North America, as published by many authors.

Efficacy Assessment

The efficacy was mostly based on findings from at least three RCTs that found a statistically significant (p<0.05) reduction in AAD incidence. The strength of evidence was determined by subtracting the number of RCTs with a non-significant outcome from the total number of RCTs with a significant outcome for an AAD-specific probiotic species. A strong strength of evidence has at least two net positive RCTs, while a moderate strength of evidence has at least one net positive RCT and zero or negative for a weak strength of evidence. The second criterion was based on the presence or absence of any adverse effects associated with a particular training therapy.

Results

General Description of the Results 

**Figure 1:**
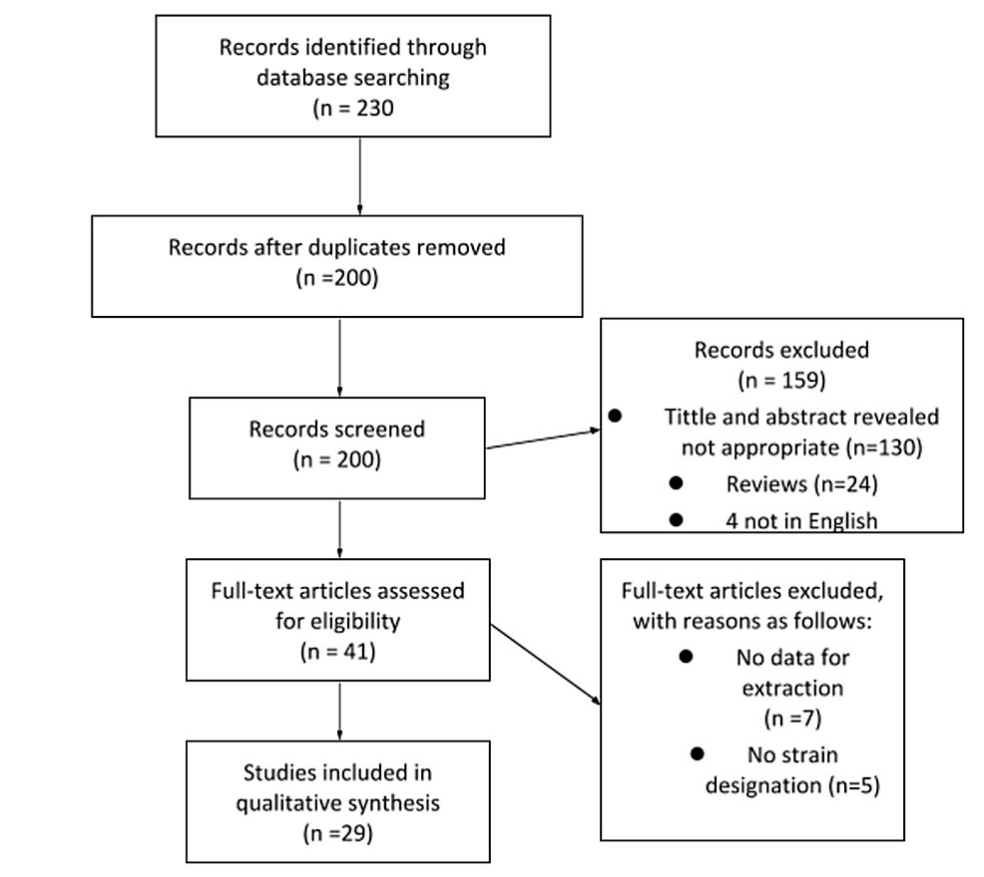
PRISMA (Preferred Reporting Items for Systematic reviews and Meta-analyses) As explained by Hutton et al. [29]

The literature search identified 230 articles that met the search criteria. Figure 1 shows the PRISMA (Preferred Reporting Items for Systematic Reviews and Meta-analyses) flow diagram for an overview of the study selection process and various reasons for the exclusion of articles as described by Hutton et al. [29]. A total of 41 articles were assessed for eligibility. Of those, 12 were excluded due to the inability to designate a specific probiotic strain (n=5) or the inability to provide the relevant data for extraction (n=7). After the screening, 29 eligible full-text articles were included in the study for qualitative synthesis.

Evidence Table 1 shows a summary of the characteristics of the eligible studies from 2010 to 2019. About ten of the eligible full-text articles were meta-analyses (evidence level 1), five were random controlled trials (RCTs; level 1), one was a non-randomized prospective cohort (evidence level 2), four were cohort and case-control studies (evidence level 3), two were prospective cohort studies with no controls, and one was a case report (evidence level 4). There were four review articles and four meta-analyses/RCTs that were not included in the evidence table but appeared in the Introduction section of this paper. The trials were conducted almost equally in both pediatric and adult populations. Almost all the studies assessed outcomes using the 95% confidence interval (CI) (p=0.05).

Table 2 shows a summary of the changing taxonomy over time of the most commonly used and studied probiotic strains in North America, as summarized by McFarland et al. [9]. The most commonly indicated Lactobacillus strains for the prevention of AAD are currently named *L. rhamnosus* GG/ATCC 53103 (*L. rhamnosus* GG in short), *L. casei* CNCM I-1518/DN114-001 (*L. casei* DN in short), and *L. acidophilus* CL1285 (*L. acidophilus* CL). These strains are often known by their brand names, such as Culturelle for *L. rhamnosus* GG, Actimel for *L. casei* DN, and Bio-K for *L. acidophilus* CL. The most commonly indicated Saccharomyces strain for the prevention of AAD is currently termed *Saccharomyces boulardii* CNCM I-745/ATCC 74012 (*S. boulardii* CNCM) and is known by its brand name as Florastor. Lastly, the most commonly indicated Bifidobacterium probiotic strain for the prevention of AAD is named *Bifidobacterium clausii* (*B. clausii*), and its common brand name is Enterogermina [10].

**Table 2:** The shifting taxonomy of probiotic strains ^a^Brand names may vary by country or formulation, most common brand name given (-), no brand name. ATCC: American Type Culture Collection, Manassas, VA, USA; CBS: Central Bureau voor Schimmelcultures, Baam, The Netherlands, CNCM: Collection Nationale de Cultures de Microorganismes (Institut Pasteur, Paris, France); DSM: Deutsche Sammlung von Mikroorganismen, Braunschweig, Germany; nr, not reported [4,9].

Probiotic brand name^a^	Older designations	Current designations
Actimel	*L. casei* immunitas *L. casei* Defensis	*L. casei* CNCM I-1518 (DN114-001)
Activia	*B. lactis* or *B. lactis* Regularis	*B. animalis* spp. Lactis DN173010 (CNCM I-2494)
Bio-k+	*L. acidophilus* CL1285 and *L. casei* LBC80R	*L. acidophilus* CL1285 and *L. casei* LBC80R and *L. rhamnosus* CLR2
Culturelle	*L. rhamnosus* GG	*L. rhamnosus* GG (atcc53103)
Dicoflor	*L. rhamnosus* GR1 and *L. fermentum* RC14	*L. rhamnosus* GR1 and *L. reuteri* RC14
Florastor	*Saccharomyces cerevisiae* boulardii *S. boulardii* lyo *S. boulardii* hansen CBS-5926	*S. boulardii* CNCM I-745 (ATCC 53103)
Ganeden BC	L. sporogenes	*B. coagulans* GBI-30, 6086
Lacidofil	*L. rhamnosus* R11 or LB24 and *L. acidophilus* R52 or YS or K1, or K300	*L. rhamnosus* R11 (CNCM I-1720) *L. helveticus* R52 (CNCM I-1722)
Lactinex	*L. bulgaricus* and *L. acidophilus*	*L. helveticus* (ATCC 33409) and *L. gasseri* (ATCC 4962)
Probi AB Oor ProViva	L. plantarum	*L. plantarum* 299v (DSM 9843)
Protecflor	*B. longum* RW001 and *L. rhamnosus* R11 and *L. accidophilus* R52 and *S. boulardii*	*B. longum* R175 (CNCM I-755) and *L. rhamnosus* R11 (CNCM I-1720) and *L. helveticus* R52 (cncm i-1722) AND *S. cerevisiae* boulardii (CNCM I-1079)
Protectis	*L. reuteri* DSM 55730 or *L. reuteri* SD2112 or *L. reuteri* ATCC 55730	*L. reuteri* DSM17938 or ATCC7938 (daughter strain)
Yakult	*L. casei* YIT9029	*L. casei* Shirota
VSL#3	*B. longum* DSM24736, *Bifidobacterium infantis* SD5220/DSM24737, *B. breve* DSM24732, *L. acidophilus* DSM247305, *L. plantarum* DSM24730, *L. paracasei* DSM24733, *L. delbrueckii* spp DSM24734, *Streptococcus thermophilus* DSM24731	*B. longus* BL03, *B. infantis* spp. Lactis BI04, *B. breve* BB02, *L. acidophilus* BA05, *L. plantarum* BP06, *L. paracasei* BP07, *L. helveticus* BD08 *Streptococcus thermophilus* BT01
-	Streptococcus faecalis	Entercoccus faecalis
-	*L. acidophilus* La-1	*L. johnsonii* ATCC 33200
-	*B. infantis* 35624	*B. longum* spp. Longum 35624
-	*B. lactis* Bb12 or *B. lactis* DSM15954	*B. animalis* spp. Lactis Bb12 (CNCM 3446)

Detailed Description of the Probiotic Strains

In general, all of the most common strains of the three genera of Lactobacillus, Bifidobacterium, and Saccharomyces showed a significant reduction in the incidence of AAD [11,12].

Lactobacillus strains: The role of the Lactobacillus genus in the prevention of AAD has been vastly studied, and the genus has produced the highest number of probiotic strains not only for the prevention of AAD but also for other most common probiotic uses [13]. Of the Lactobacillus genus, *L. rhamnosus* GG, *L. casei* DN, and *L. acidophilus* CL were the most efficacious strains, according to findings from a meta-analysis by Cai et al. [8], confirming previous findings from a meta-analysis by Ripert et al. [10], indicating that probiotic formulations containing these three Lactobacilli strains (*L. acidophilus* CL, *L. casei* DN, and *L. rhamnosus* GG) are the most efficacious probiotic combination.

For *L. rhamnosus* GG, a meta-analysis of 228 trials by McFarland et al. [9] pointed out that all pooled RCTs for *L. rhamnosus* GG demonstrated significant efficacy only for the pediatric AAD and not for the other indications. Several other authors have supported this same view. The latest meta-analysis by Sniffen et al. [14] showed that there were only four RCTs with significant evidence for AAD prevention from *L. rhamnosus* GG therapy versus six RCTs that did not show any significant evidence, for a net negative (-2) RCT score. One meta-analysis by Blaabjerg et al. [15] contrasted this position, arguing that *L. rhamnosus* GG does not only prevent AAD in pediatric patients but also prevents it in adults, since they found the incidence of AAD in only 8.0% of the probiotic group of combined *L. rhamnosus* GG and *S. boulardii*, compared to 17.7% in the control group (RR 0.49, 95% CI 0.36 to 0.66). The reported adverse effect associated with *L. rhamnosus* GG treatment was nausea [8].

Focusing on *L. acidophilus* CL, an RCT by Sampalis et al. [13] that focused particularly on *L. acidophilus* CL demonstrated that the incidence of AAD was only 21.8% in Bio-K + CL1285 (that is a brand name for *L. acidophilus* CL) subjects versus 29.4% in the placebo group, with the adjusted odd ratio (OR) being 0.627 at p=0.037, showing significant efficacy of *L. acidophilus* CL. Regardless of this evidence, in addition to the evidence given by Cai et al. [8] above, the meta-analysis by Sniffen et al. [14] showed that there were only four RCTs with significant evidence for AAD prevention from *L. acidophilus* CL therapy versus six RCTs that did not show any significant evidence, for a net negative (-2) RCT score (weak), similar to the *L. rhamnosus* GG findings. According to Sampalis et al. [13], the adverse outcomes of the *L. acidophilus* CL therapy were nausea, flatulence, and constipation, which were reported by 72% of both the Bio-K+ and the control groups. According to Sniffen et al. [14], only *L. casei* DN showed strong evidence with two positive RCTs versus a null RCT that did not show any significant evidence.

Finally, the strongest and latest evidence for the prevention of AAD by *L. casei* DN was presented in Alberta (Canada) hospital research, as documentation of AAD in only 12.5% of the RCT probiotic group versus 31.3% in the control group was made [16]. An RCT carried out by Dietrich et al. [17], comparing *L. casei* DN versus *L. shirota*, showed that AAD incidence was significantly reduced in the intervention group (6.7% versus 33.3%; p<0.021) that had the *L. casei* DN than in the group with the *L. casei* Shirota. The adverse effect associated with the *L. casei* DN administration was instant emesis [16].

Saccharomyces strains: *Saccharomyces boulardii* I-745 (*S. boulardii* in short), a fungal strain, has strong evidence for the prevention of both adult and pediatric AAD and traveler’s diarrhea [14,18]. Sniffen et al. [14] found 18 RCTs that showed significant evidence for AAD prevention versus nine RCTs that did not show any significant evidence for this fungal strain. A meta-analysis by McFarland [18] found that eight out of ten (80%) RCTs showed significant efficacy for the prevention of AAD, with a pooled relative risk of 0.47 at a 95% CI of 0.35-0.6. McFarland [19] also pointed out that randomized *H. pylori* patients to placebo, *L. rhamnosus* GG, *S. boulardii*, *L. acidophilus* CL, and *Bifidobacterium lactis* demonstrated significant efficacy for the prevention of AAD in only the *S. boulardii* treatment group. He did not find any other strains of Saccharomyces that demonstrated significant efficacy for the prevention of AAD. An adverse outcome related to *S. boulardii* therapy was pointed out in a case report by Thygesen et al. [20], finding fungemia in immunocompromised patients concurrently treated with both antibiotics (vancomycin) and the *S. boulardii* probiotic known as Sacchaflor.

Bifidobacterium strains: According to Cai et al. [8], *B. clausii* is the most efficacious Bifidobacterium strain and one of the three most commonly indicated probiotics for AAD prevention. Few trials/meta-analyses have been carried out to investigate the efficacy of *B. clausii* for the prevention of AAD in North America, yet according to Yun et al. [21], Bifidobacteria are major gastro-intestinal commensal microbes, "comprising up to 90% of all bacteria in fecal samples of breast-fed infants." Much of the claim for the efficacy of Bifidobacterium in the prevention of AAD comes from India and Pakistan [22]. Patrone et al. [22] pointed out that *B. clausii* products are sold under four brand names in India and Pakistan: Tufpro, Ecogro, Enterogermina, Entromax, and Ospor, with Enterogermina being the most common. Patrone et al. [22] also pointed out that only Enterogermina demonstrated some scientific evidence for their label claims, while the other three did not.

Lakshmi et al. [23] demonstrated significant efficacy in *B. clausii* use against AAD and its safety. Another RCT by Chatterjee et al. [24] showed an AAD incidence in only 10.8% of the group randomized to a mixture of Bifidobacterium spp. and *L. acidophilus* CL, compared to 15.6% in the placebo group (RR: 0.7; 95% CI: 0.4-1.2). In contrast to these claims of *B. clausii* usage to prevent AAD, Yun et al. [21] pointed out that Bifidobacteria probiotics are active against *C. difficile*-associated diarrhea (CDAD) and often indicated for its therapy rather than AAD, particularly *B. longus* ATCC 15707, supporting a similar previous finding by Valdés-Varela, Hernández-Barranco et al. [25]. A screening of 20 strains of Bifidobacteria and Lactobacilli by Valdés-Varela et al. [26] demonstrated that most Bifidobacterium strains showed significant efficacy towards CDI prevention rather than AAD, a finding they thought was quite opposite to the Lactobacillus strains.

Mixed strains: Table 3 shows a summary of the recommendations for the use of probiotics, particularly in childhood intestinal diseases, by geographic region, as provided in a meta-analysis by Cameron et al. [27]. Of utmost importance to this review is the row that outlines the recommended antibiotics for the prevention of AAD. The USA, Europe, and Latin America recommend only *S. boulardii* and *L. rhamnosus* GG. In addition to the above two strains, the rest of the world's regions also recommend a mixture of *B. lactis* Bb12 and *Saccharomyces thermophiles*, and *L. rhamnosus* strains E/N, Oxy, and Pen. Recommendations from the USA researchers as presented in the meta-analysis by Sniffen et al. [14] are, however, slightly different than those pointed out by Cameron et al. [27] in Table 3. Sniffen et al. [14] recommended *S. boulardii* I-745, *L. casei* DN114001, and LaLcLr mix (a combination of *L. acidophilus* CL1285, *L. casei* Lbc80r, and *L. rhamnosus* CLR2) for AAD prevention in any age group. Cameron et al. [27] pointed out a significant factor that may immensely affect the recommendation for a specific probiotic strain by geographic region: some probiotic strains are restricted to a specific climatic region. For instance, *L. rhamnosus* GG is not widely common in Japan, yet *L. casei* Shirota is widely available [27].

**Table 3:** Recommendations for use of probiotics by geographic region ^1^Available evidence supports use in UC but not CD or pouchitis; ^2^For mildly active UC. T: treatment; P: prevention; AAD: antibiotic-associated diarrhea; CDAD: *Clostridium difficile*-associated diarrhea; CD: Cronhn's disease; ibd: Inflammatory bowel disease; IBS: Irritable bowel syndrome; UC: ulcerative colitis; VSL#3: Proprietary mixture of eight probiotic strains. Figure modified from Cameron et al. [27].

Diseases	Indication	Europe	USA	Latin America	World
Acute gastroenteritis	T	*L. rhamnosus* GG, *S. boulardii*, *L. reurteri*	*L. rhamnosus* GG, *S. boulardii*	*L. rhamnosus* GG, *S. boulardii*, *L. reuteri*	*L. rhamnosus* GG, *S. boulardii*, Indian Dahi
AAD	p	*L. rhamnosus* GG, *S. boulardii*	*L. rhamnosus* GG, *S. boulardii*	*L. rhamnosus* GG, *S. boulardii*	*L. rhamnosus* GG, *S. boulardii*, *B. lactis* Bb12 + *S. thermophilus*, *L. rhamnosus* strains E/N, Oxy and Pen
CDAD	P	S. boulardii			
Nosocomial diarrhea	P	*L. rhamnosus* GG	*L. rhamnosus* GG	*L. rhamnosus* GG, *B. lactis* Bb12, *S. thermophilus*, *B. bifidum*	*L. rhamnosus* GG, *B. lactis* Bb12 + *S. thermophilus*
Traveler’s diarrhea	P			S. boulardii	
Functional intestinal disorders (IBS)	T	Insufficient evidence		*L. rhamnosus* GG, VSL#3	*L. rhamnosus* GG, l. REUTERI dsm 17938
Infant colic	T	*L. retuteri* DSM 17938		*L. reuteri* DSM 17938	*L. reuteri* DSM 17938
IBD (CD, UC, pouchitis)	T	*E. coli* Nissle 1917, VSL#3^1^		VSL#3^1^	VSL#3^2^
*Helicobacter pylori* infection	T			Not recommended	*L. casei* DN-114 001

Discussion

In this systemic review, direct and indirect evidence was derived from 29 articles published in peer-reviewed journals, comprising mostly meta-analyses, RCTs, observational studies, and a few reviews that analyzed the relative efficacy of strains of the three most commonly used probiotic genera to prevent antibiotic-associated diarrhea. In general, the most common strains of the three genera of Lactobacillus, Bifidobacterium, and Saccharomyces showed significant efficacy in the prevention of antibiotic-associated diarrhea [12]. Several other specific observations for each of the efficacious strains were also made. The three most efficacious Lactobacillus probiotic strains were *L. rhamnosus* GG, *L. casei* DN, and *L. acidophilus* CL [8]. *L. rhamnosus* GG seems to be only efficacious in pediatric patients, while *L. casei* DN tends to be effective for both pediatric and adult AAD patients. The most efficacious strain of Saccharomyces for both pediatric and adult patients was *S. boulardii*, while the most efficacious strain of Bifidobacteria for both adult and pediatric patients is *B. clausii*, although *B. clausii* therapy is most often associated with instant emesis.

Before expanding on the findings in this review, there were several mechanisms of AAD prevention by probiotics that were pointed out by various authors. Song et al. [28] outlined that Lactobacillus strains prevent AAD through competition for nutrients such as N-acetyl-glucosamine and sialic acid in the intestines. Sampalis et al. [13] thought that Lactobacillus strains prevent AAD through the modulation of intestinal cytokine production, especially through the suppression of tumor necrosis factor-alpha (TNF-a) and interleukin-8 (IL-8) production by the T cells. The probiotics also elevate the synthesis of short-chain fatty acids, reducing the immune response [13]. Ripert et al. [10] noted that *S. boulardii* and *B. clausii* protect the host from pathogenic intestinal infection through enzymatic removal or cover of the mucosal epithelial cells. Ripert et al. [10] also pointed out that most probiotics produce inhibitory compounds such as acetate, propionate, butyrate, H_2_O_2_, and bacteriocins, reducing infection from pathogenic microorganisms.

To shed light on the findings from the three Lactobacillus strains that showed significant efficacy for the prevention of antibiotic-associated diarrhea, the finding by Blaabjerg et al. [15] that *L. rhamnosus* GG prevents both adult and pediatric AAD may not be necessarily true. This is because the authors combined both the *L. rhamnosus* GG and the *S. boulardii* strains in the treatment group. The significant evidence they found in adult patients may be solely attributed to *S. boulardii* as opposed to *L. rhamnosus* GG. This means that the use of *L. rhamnosus* GG to treat AAD may only be limited to pediatric patients, as found by many authors, including McFarland et al. [9]. Although Cai et al. [8] and Sampalis et al. [13] found significant efficacy for *L. acidophilus* CL strain use to prevent AAD, the contrasting net negative score (four RCTs supporting its efficacy versus six RCTs opposing its efficacy) by Sniffen et al. [14] makes the use of this strain controversial. Even more controversial is the fact that a whopping 72% of the patients randomized to *L. acidophilus* CL experienced flatulence, nausea, and constipation [13]. *L. casei* DN used to prevent AAD seems to be the most promising of the three Lactobacillus strains, given the significant efficacy findings from Alberda et al. [16] and Dietrich et al. [17]. The only concerning contraindication for *L. casei* DN is instant emesis [16].

For the Bifdiobacterium strain, *B. clausii*, the foremost concern is the lack of evidence for its efficacy to prevent AAD across the globe. There is hardly any RCT on it in North America, with the only evidence for it coming from Southeast Asia and Far East Asia [21,22]. If 90% of all intestinal bacteria comprised Bifidobacteria, as claimed by Yun et al. [21], then much attention would have probably been focused on it if it indeed prevented AAD. Even more concerning is the fact that an investigation by Patrone et al. [22] pointed out that among the four band names for *B. clausii* in patients and Pakistan, only Enterogermina followed the label claims. Findings from Valdes-Varela et al. [26], Valdes-Varela et al. [25], and Yun et al. [21] blandly opposed the efficacy of *B. clausii* for the prevention of AAD altogether, rather than supporting its exclusive indication to treat *C. difficile* infection.

Looking at the strains from Saccharomyces, perhaps the most promising probiotic strain to prevent AAD in both pediatric and adult patients is *S. boulardii* I-745. The net number of RCTs for its efficacy to treat AAD was nine (18 RCTs showed significant evidence versus 9 RCTs that showed non-significant evidence), the strongest evidence compared to any other probiotic strain for a similar indication [14]. This strength of evidence built upon a previous strong proof from McFarland [19], who found that eight out of 10 (80%) RCTs showed significant efficacy for the prevention of AAD, with a pooled relative risk of 0.47 at a 95% CI of 0.35-0.6. The only adverse outcome found by Thygesen et al. [20], that an *S. boulardii* brand known as Sacchaflor caused fungemia in immunocompromised patients, may be true, but this may be true for any probiotic strain, if ever investigated, since immunocompromised individuals are generally susceptible to any foreign microbe.

Lastly, the regional recommendations for the use of probiotics by geographic region as presented by Cameron et al. [27] and Sniffen et al. [14] tend to conflict with the efficacious indications for AAD prevention, especially in the North American region. While the recommendations from Sniffen et al. [14] do not include *L. rhamnosus* GG for AAD prevention in any age group, Cameron et al. [27] recommend this strain for the pediatric age group, supporting the stand of many other authors. Cameron et al. [27] mention rare probiotic strains such as *B. lactis* Bb12, *S. thermophiles*, and *L. rhamnosus* strains E/N, Oxy, and Pen as the recommended strains for AAD prevention in a few countries around the world. The perceived relatively increased efficacy of these rare probiotic strains probably has to do with the climatic restrictions on the availability of the most commonly used probiotic strains from the three most commonly used probiotic genera worldwide.

Given these revelations from the strains of the three most commonly used probiotic genera to treat AAD, it is safe to rank the efficacy of these strains. *S. boulardii* has the strongest evidence for the treatment of both adult and pediatric AAD patients, while *L. casei* DN ranks second for a similar age group. *L. acidophilus* CL may occupy the third rank, subject to further investigation of efficacy evidence, while *B. clausii* should probably never be categorized with the strains that prevent AAD; rather, it should be categorized with the strains that are used to manage CDI. If dealing with pediatric cases, *L. rhamnosus* GG should probably be the number one choice for the prevention of AAD. These rankings unfortunately disapprove the hypothesis posted earlier in this review that a Lactobacillus strain is the most efficacious for the prevention of antibiotic-associated diarrhea. The hypothesis was derived from the many misinformed review articles out there that mostly single out *L. rhamnosus* GG as the most commonly used probiotic strain for AAD prevention. These rankings assume that similar dosages and formulations, that is, yogurt or capsules, among others, are used.

These observations should, however, be taken with some caution, given the many limitations that were prominent in this literature review. First off, the restrictions imposed on retrieved articles could change these findings in so many ways. For instant, only full-text articles that were freely available were investigated. Contrasting evidence may be found in the articles that had to be paid for but were not investigated in this review. Also, the exclusion of non-English-translated articles excluded high-quality articles on the subject matter. These restrictions are especially concerning given the limited availability of RCTs for specific strains indicated for a particular disease.

Another limitation is the changing taxonomy of bacterial and fungal species for strain designations, and even more confusing is the lack of a global consensus on strain designations [4]. To overcome these two limitations, most trials and meta-analyses did not reveal the specific strain name and instead used the genus names. Consequently, studies that used vague probiotic strain names were excluded from this review, greatly affecting the findings.

Last but not least, the observations and rankings made in this review are qualitative. A pair-wise comparison is probably needed to quantitatively rank the relative efficacy of the probiotic trains that are indicated for AAD prevention. Also, pooled relative risk ratio studies may be required to further quantitatively find the true relative efficacy of the most commonly used probiotic strains. These quantitative variables should preferably be computed with larger sample sizes than those presented in most of the articles used in this review.

## Conclusions

Unfortunately, much of the current literature inappropriately pools together probiotic strains into broad categories such as "Bifidobacterium strains" without accounting for strain specificity for a specific disease indication. Though a few papers do account for the probiotic strain specificity needed to treat a particular disease, they do not account for the relative efficacy of the specific strains. This paper goes a step further by comparing the relative efficacy of the strains of these three most commonly indicated probiotic genera to treat antibiotic-associated diarrhea: Lactobacillus, Bifidobacterium, and Saccharomyces.

The choice of the best probiotic for AAD prevention will continue to be a shifting target, given the ever-changing taxonomy of nomenclature and as more clinical trials are done. Insights from this paper point out some specific strains, as far as state-of-the-art research is concerned, that a patient or a healthcare practitioner should look out for in any given probiotic formation and dosage. Whenever probiotics are considered for the prevention of AAD in both pediatric and adult patients, *S. boulardii* I-745 should probably be the first line of management. If that does not help, *L. casei* DN should be the next in line, and if those two fail, then *L. acidophilus* CL should be the last resort. These findings disapprove the hypothesis of this review that a Lactobacillus strain is the most efficacious for the prevention of antibiotic-associated diarrhea.
